# Examining partnerships within an international knowledge translation network focused on youth mental health promotion

**DOI:** 10.1186/s12961-020-0535-x

**Published:** 2020-03-04

**Authors:** T. Halsall, I. Manion, J. Henderson, P. Robeson, R. Purcell, P. Liversidge, S. N. Iyer

**Affiliations:** 1 Youth Research Unit, The Royal’s Institute of Mental Health Research, 1145 Carling Ave., Ottawa, Ontario K1Z 7K4 Canada; 2 Margaret and Wallace McCain Centre for Child, Youth and Family Mental Health, Centre for Addiction and Mental Health, Toronto, Ontario Canada; 3 University of Toronto, Toronto, Ontario Canada; 4 Children’s Healthcare Canada, Ottawa, Ontario Canada; 5 Orygen, Parkville, Victoria Australia; 6 Centre for Youth Mental Health, The University of Melbourne, Parkville, Victoria Australia; 7 Alberta Integrated Youth Services Initiative, Edmonton, Alberta Canada; 8 ACCESS Open Minds (pan-Canadian youth mental health research network), Montreal, Quebec Canada; 9 Douglas Mental Health University Institute, Montreal, Quebec Canada; 10 Department of Psychiatry, McGill University, Montreal, Quebec Canada

**Keywords:** Integrated youth services, youth mental health, knowledge translation, collaboration, social network analysis, system transformation

## Abstract

**Background:**

Systems transformation for health promotion, involving engagement from multiple disciplines and levels of influence, requires an investment in partnership development. Integrated youth service is a collaborative model that brings organisations together to provide holistic care for youth. Frayme is an international knowledge translation network designed to support the uptake and scaling of integrated youth service. Social network analysis (SNA) is the study of relationships among social units and is useful to better understand how partners collaborate within a network to achieve major objectives. The purpose of this paper is to apply SNA to the Frayme network in order to (1) examine the level and strength of partnerships, (2) identify the strategies being employed to promote the main objectives and (3) apply the findings to current research in youth mental health and system transformation.

**Methods:**

The PARTNER tool includes a validated survey and analysis software designed to examine partner interconnections. This tool was used to perform the SNA and 51 of the 75 partners completed the survey (14 researchers, 2 advisory groups and 35 organisations). A network map was created and descriptive frequencies were calculated.

**Results:**

The overall network scores for the Frayme network were 20.6% for density, 81.5% for centralisation and 71.7% for overall trust. The Frayme secretariat received a 3.84 out of a possible 4 for value. In addition, the youth and family advisories each received a value score of 4 and all Leadership Team organisations received a score of 2.97 or above.

**Conclusions:**

The Frayme secretariat links many partners who would otherwise be disconnected and acts as a significant conduit for novel information. Frayme may have the opportunity to enhance value perceptions among broader network members by profiling individual organisations and the potential leveraging opportunities that might exist through their work. These findings increase understanding with respect to the mechanisms of network development and will be helpful to inform partnership development in the future. In addition, they contribute to the literature with respect to knowledge translation practice as well as the scaling of collaborative interventions within youth mental health.

## Introduction

Systems transformation for health promotion, involving engagement from multiple disciplines and levels of influence, requires an investment in partnership development [1, 2]. These collaborations function by leveraging resources, combining skill sets and addressing health issues that result from a range of factors [3]. In addition, researchers have argued that the analysis of social connections within networks is the most valuable way to reveal the smaller scale interactions that influence broad scale patterns [4]. Integrated youth service (IYS) is a collaborative model that brings organisations together to provide holistic care that often includes mental health, substance use and addictions, primary care (including sexual health) and other social services [5–9]. Frayme is an international knowledge translation (KT) network designed to support the uptake and scaling of IYS [5, 10]. This paper examines the partnerships developed within the Frayme network using social network analysis (SNA) and applies the findings to current research in KT for health promotion in order to enhance understanding of how such networks can improve outcomes in youth mental health.

### IYS and Frayme

In Canada, the mental health system is characterised by segmented, specialised services designed to target isolated problems that lack a consideration of contextual determinants on mental health [5]. As a result, services are unable to provide holistic support that can be tailored to the individual within their context. IYS models were designed to overcome these issues through establishing and/or formalising collaborative relationships among community-based providers that link services and specialisations to create a comprehensive offering of supports. IYS models support public health approaches in that they involve collective efforts that extend beyond individual services or programmes. In addition, recognising that the majority of mental disorders commence before adulthood [11] and that early intervention for mental health issues has been associated with better outcomes [12–14], they align with a focus on prevention while taking a comprehensive approach. Although there is a large variation among models, they commonly incorporate a general set of components that include stepped-care approaches, evidence-based interventions (such as solution-focused brief therapy, cognitive behavioural therapy and strengths-based approaches), youth and family engagement, adapted policies to support inter-organisational collaboration and a governance structure that oversees the overall partnership [10]. Stepped care models typically involve multiple levels of treatment intensity and clients are offered interventions at the most appropriate and least intensive level based on an assessment. Going forward, interventions can be stepped up or down the levels based on clients’ illness/problem progression and needs. Youth and family engagement within IYS recognises the key importance of lived experience and maintains a focus on this lens when designing and delivering services. Although models vary, integrated youth services often combine a range of youth- and family-centred approaches such as peer support, technology-enhanced services and accessible physical spaces.

“*Above all, the development of IYS requires the building of positive relationships*” ([10], p. 52). Capitalising on existing networks and leveraging on-going initiatives, Frayme was established in the late spring of 2017 to support the creation of IYS partnerships and to enhance uptake of IYS services through knowledge mobilisation and implementation supports. Currently, Frayme has developed linkages with 370 partners working within youth mental health systems in 9 Canadian provinces and 13 countries across the world (Australia, Austria, Canada, Denmark, France, Hong Kong, Ireland, Norway, New Zealand, Sri Lanka, Sweden, the United Kingdom and the United States). Partners include researchers, consumer advocates, practitioners, policy-makers and relevant national and international networks that are working within a range of disciplines, including psychology, health administration, psychiatry, education, social work and public health, among others.

At the outset, the Frayme network was awarded funding from the Networks of Centres of Excellence – International Knowledge Translation Platforms competition along with significant support from key partners [10]. KT in the healthcare context has been defined as a process that ensures “*stakeholders are aware of and use research evidence to inform their health and healthcare decision making*” ([15], p. 2). Frayme’s approach to knowledge translation combines knowledge synthesis, knowledge mobilisation and implementation science in order to enhance current understanding of best practices within integrated youth services and to support uptake and scaling [16]. Frayme’s integrated knowledge mobilisation approach is based on the Co-produced Pathway to Impact [17], which is a logic model adapted to illustrate the collaborative engagement between knowledge users and producers across the stages of knowledge creation, dissemination and uptake. Frayme places an emphasis on youth and family engagement as well as research and practice evidence, and incorporates key insights from each of these stakeholder groups to inform strategy and focus. This is accomplished through the creation of the Advisory on Youth Matters (AYM) and a family advisory as well as the representation of youth and family perspectives on the Board of Directors. These individuals provide strategic guidance to support Frayme objectives as well as to inform operational processes.

### Partnership in Knowledge Translation

Partnership development is considered a significant component within KT efforts [18, 19] and this may include formal relationships, such as collaboration in a grant as well as more informal interactions such as sharing advice or making strategic introductions [19]. Researchers have highlighted the importance of interconnections between researchers and clinicians [18] as well as the importance of the exchange of health information with policy-makers, funders, consumers and their families [15]. In the case of consumers, KT can help support health decision-making and enhanced capacity for self-care [15].

SNA is the study of relationships among social units, such as individuals, groups or organisations [20, 21], and the influence on the overall network [22]. Hawe [20] argues that, as researchers continue to recognise the multiple contexts of influence on health, there will be an increased application of SNA in order to understand network solutions. There are a variety of characteristics and measures that can be used to describe networks such as density (the total number of relations) or centrality (identifies the more closely connected network members) [20]. Dense networks have the capacity for stronger coordination of activity as many partners are inter-connected [20]. However, Varda [22] argues that more connections are not necessarily better. She suggests that the practice of counting partner numbers can help to support stakeholder buy-in; however, a stronger approach may involve an examination of the quality of interactions and the strategic connections that help to advance the overall mission of the network [22, 23]. In addition, direct measurement of the frequency of communication may not be a valid indicator of enhanced partnership, as established relationships may not necessitate frequent interactions [23]. This has been demonstrated in KT networks, whereby strategic recruitment of new members supported interdisciplinary research collaborations and broader impact [19].

There is an expanding literature on SNAs that are conducted within KT initiatives and a recent scoping review was conducted to examine how SNA can be applied to further our understanding of KT among health professionals [24]. The authors found that SNA was useful for examining network features that relate to knowledge mobilisation, implementation and the uptake of evidence [24]. SNA can also highlight underlying mechanisms that create impacts through social relationships within KT networks [24]. For example, SNA can help to interpret the influence of knowledge brokers and the interactions among subgroups such as tendencies to exchange with similar others and the role of social hierarchies [24].

In a KT network designed to enhance cancer care that was supported by a network director, manager and small operational support staff, a SNA identified that network membership increased from 68 to 244 over 4 years and 40% of the connections were created between members who did not have previous relationships [19]. In addition, one-third of survey participants noted that they were involved in projects that were not directly funded by the KT network, but that were the result of partnerships that were created through network membership and three-quarters of respondents described changes in their practice that had resulted through participation in the network. Researchers have identified that knowledge brokers can also successfully grow stakeholder networks in the pursuit of increasing uptake of scientific evidence [25]. In addition, they can enhance stakeholder understanding of the knowledge needs of other network members, including the nature of operating environments allowing for more focused delivery of knowledge exchange [25]. Knowledge brokers may also help to overcome partnership challenges related with crossing interdisciplinary boundaries that result from the highly specialised nature of many disciplines and hegemonic variation among collaborators [19].

SNA can also support the examination of patterns of inter-relationships within KT networks in order to support network leaders in managing partner interactions and develop strategic partnerships [18]. There are several factors that have been identified that tend to be related with increased interactions within KT networks, including geographic proximity, homophily (professional similarity) and past relationships [18, 19]. With respect to centrality within KT networks, researchers have identified that more centralised networks, wherein partners are arranged as spokes around a network manager ‘hub’, can maintain stronger control and standardisation over the messages being disseminated [26]. In contrast, a more distributed leadership approach allows for more opportunities for innovative theory and idea generation [26].

### Purpose

There is a need for more research examining how networks promote health impacts [1, 3, 23, 27]. Information with respect to network relationships can be used to inform strategic development and to identify mechanisms that support change and accountability [3]. As such, the purpose of this paper is to apply SNA to the Frayme network in order to (1) examine the level and strength of partnerships, (2) identify the strategies being employed to promote the main objectives and (3) apply the findings to current research in youth mental health and system transformation. These results will serve as an indicator of partnership and network effectiveness, and may support efforts to enhance the engagement of fellow Frayme collaborators [1]. The findings will also be useful for other network managers working in interdisciplinary initiatives to promote KT [19].

## Methods

### Sample

In this case, the network we were examining included organisations, individual researchers and advisory groups in Canada and abroad that are part of the Frayme network. The sample was created from a bounded network [3], whereby a list of participants was generated from the broader network of Frayme partners. This list was developed through a discussion with the network’s Scientific Director, the Director of Operations and the first author. Partners were included if they had been involved in the Frayme network sufficiently to be familiar with the mission of the network and to have some knowledge of the work that had been accomplished. The final list included 75 partners. The main contact from each of the organisations was invited to complete an online survey. Ethics approval was obtained from the Royal Ottawa Health Care Group Research Ethics Board and informed consent was received from all survey respondents.

### Survey

We utilised the PARTNER tool to perform the SNA [3, 22]. The PARTNER tool includes a validated survey and analysis software designed to examine partner interconnections. The survey included questions that examined respondent motivation for participating in the network, resource contributions, length of time of involvement, nature of contributions to the network, perceived level of success and major outcomes. The survey also contains relational questions, whereby respondents selected other partners from the original list of 75. These questions captured information about specific relationships among partners with respect to frequency of interaction, perceptions of trust and value, and level of collaboration. The PARTNER tool analyses this information to calculate the network density and degree of centralisation, which relates to the extent of overall partnership development and pattern of the relationships.

Descriptive analyses examined the interconnections between partners and the nature of partnership at the organisational, dyadic and network levels. Dyadic analysis captured reciprocal perceptions of relationships and organisational characteristics among collaborating network members. Whole network scores with respect to density, centralisation and overall trust were calculated by aggregating responses from across all members.

## Results

The survey was distributed in the fall of 2018 and five reminders were sent over a 3-week period. Some key partners received additional emails and prompts to complete the survey. When the survey was closed, 51 of the 75 partners (14 individuals (researchers), 2 advisory groups and 35 organisations) completed the survey for a response rate of 68%. Prior to the survey, respondent categories were created and all 75 partners were assigned to each category (Table 1). Respondent organisations whose work relates to system transformation (e.g. the Mental Health Commission of Canada and the Canadian Centre on Substance Use and Addictions) were classified as ‘System Transformation’ partners. The average length of time of involvement was 17.3 months with a range of 5 to 24 months.

**Table 1:** Network members and survey respondents by stakeholder category

	Actual network members	Survey respondents
Researcher	22	14
System transformation	17	16
Practitioner	15	8
IYS site	7	6
Policy	7	3
Philanthropy	4	2
Family	1	1
Media	1	0
Youth	1	1
Total	75	51

**Fig. 1:**
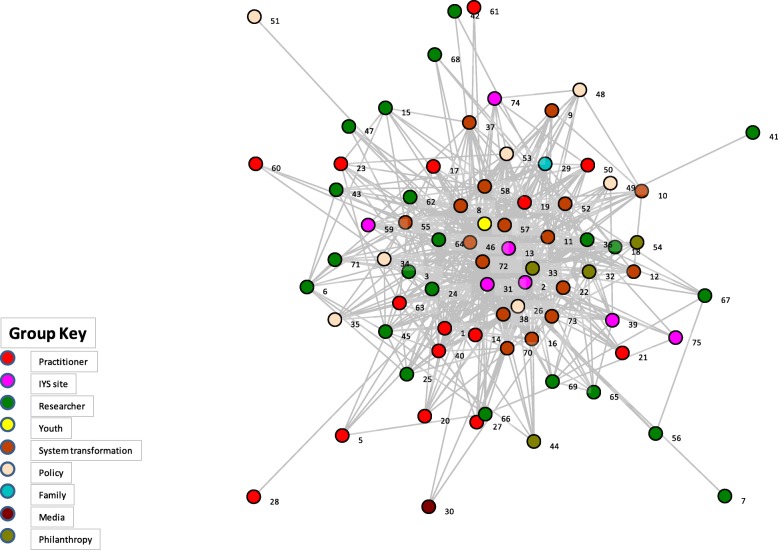
Frayme Network Map. Note: The table displays nodes that represent each Frayme partner. These are colour-coded based on stakeholder groups created to categorise Frayme partners. Lines that connect nodes indicate that, at minimum, one of the partners (nodes) identified a relationship between themselves and the other partner (node). Distances between nodes and length of lines are not significant; however, partners with more connections tend to be more centrally located in the network map

In terms of overall network scores, the density score for the Frayme network was 20.6%. This number represents the percentage of ties that exist within the network with respect to the total possible number of ties. This suggests that about one-fifth of all possible relationships have been created. The centralisation score was 81.5%, suggesting that the Frayme network is highly centralised with most connections radiating from a small subset of highly connected network members. The overall trust score was 71.7%, indicating that there was a high level of partner ratings that describe the other network collaborators as being reliable, having mission congruence with the Frayme mission, and maintaining open and transparent communication [3]. With respect to value, the Frayme secretariat (i.e. key Frayme operational staff) received a 3.84 out of a possible 4. In addition, the youth and family advisories each received a score of 4 and all Leadership Team organisations and one funder received 2.97 or above. These organisations included the Frayme secretariat, ACCESS Open Minds, the McCain Centre for Child, Youth & Family Mental Health at the Centre for Addiction and Mental Health, Youth Wellness Hubs Ontario, the Royal Ottawa Mental Health Centre, Foundry, Orygen (University of Melbourne in Australia) and the Graham Boeckh Foundation. The value score is calculated based on partner perceptions of power and influence within the network, level of involvement and resource contribution. Lastly, the Frayme secretariat had 66.39 non-redundant ties or connections that are unique conduits of information flow. Figure 1 displays the network map of the Frayme network.

### Outcomes, success and collaborative strategies

**Fig. 2:**
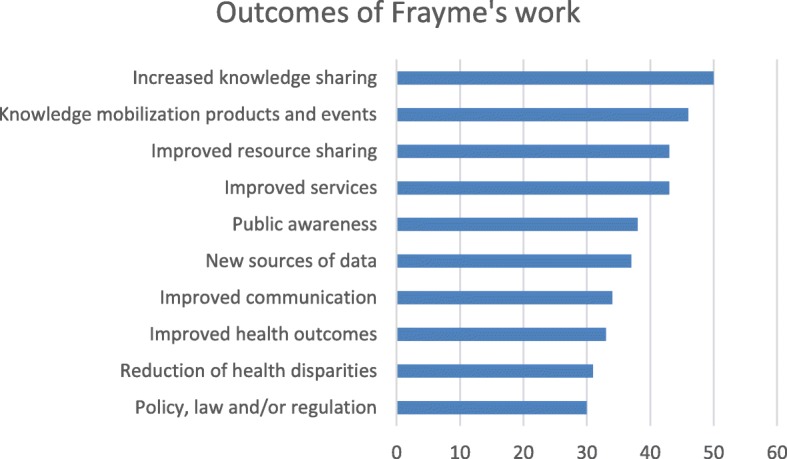
Outcomes of Frayme’s network

Frayme’s most important outcome was identified by network members as being increased knowledge sharing (50%). Other outcomes identified are listed in Fig. 2. These include increased knowledge sharing, knowledge mobilisation products and events, improved resource sharing, improved services, public awareness, new sources of data, improved communication, improved health outcomes, reduction of health disparities and policy, law and/or regulation. More frequently cited outcomes were related to short-term impacts that would be related to enhanced interactions and coordination among partners. Outcomes that were less common were often related to long-term impacts such as changes in policy, practice and health.

**Fig. 3:**
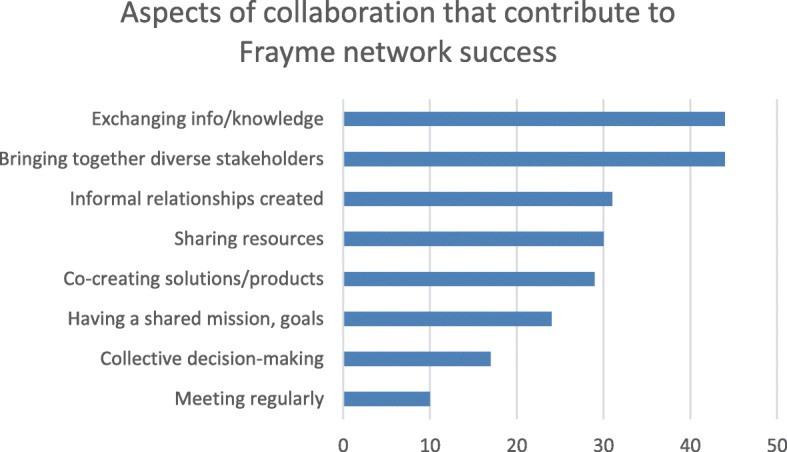
Aspects of collaboration that contribute to Frayme network success

Frayme network members also described their perceptions of the level of success that Frayme had achieved; 51% of respondents believed it was too early to tell, 43% identified that Frayme had been successful or somewhat successful, and 5.9% felt that Frayme had been very successful at achieving its goals. Figure 3 lists the collaborative strategies that partners felt contributed to this achievement. In descending order of frequency, these include exchanging info/knowledge, bringing together diverse stakeholders, informal relationships created, sharing resources, co-creating solutions/products, having a shared mission/goals, collective decision-making and meeting regularly. Some of these have overlap with identified outcomes and are a reflection of the functional aspects of the work as perceived by the partners.

### Level of collaboration

With respect to level of collaboration, there were 691 total dyads of interactions reported by partners. From among these interactions, 70 (11.43%) responses reported a relationship where there were no collaborative activities being completed, while the remaining responses described relationships as reflecting cooperative activities (335, 48.48%), coordinated activities (142, 20.55%) and integrated activities (135, 19.54%).

### Resource contribution

In terms of resource contribution, the most frequently reported resource was network connections, followed by information/feedback and content expertise. The least frequent resource reported was information technology and web resources (Table 2).

**Table 2:** Resources contributed by Frayme partners

Resources contributed by Frayme partners	Number of responses
Network connections	46
Info/Feedback	44
Content expertise (e.g. youth mental health, integrated youth services)	38
Method expertise (e.g. implementation science, evaluation)	29
Advocacy	27
In-kind resources (e.g. meeting space, staff time)	26
Facilitation/Leadership	25
Data resources including data sets, collection and analysis	15
Fiscal management (e.g. acting as fiscal agent)	4
Funding	3
IT/web resources (e.g. server space, web site development, social media)	3

## Discussion

This research used SNA to examine the relationships that were developed within an evolving KT network designed to enhance youth mental health system integration. Overall, within its first 2 years of operation, the Frayme network demonstrated a high level of trust and a generally centralised structure. Value ratings were high for network partners holding leadership roles, including organisations from the Leadership Team, a major funder and two advisories.

The Strength of Weak Ties theory [4] hypothesises that the stronger the tie (as defined by time commitment, emotional connection and reciprocity) among two actors, the higher the likelihood that they have overlapping social spheres and redundant ties to other network members. This theory also suggests that weak ties are often better conduits for information transfer between groups within networks. Within KT networks, weak ties have been conceptualised as opportunities for collaboration created through network participation whereby previous relationships did not exist [18]. The Frayme secretariat holds a special position in the network in that it has 66.39 non-redundant or weak ties from among 74 possible connections. As such, the Frayme secretariat links many partners who would otherwise be disconnected and, based on the Strength of Weak Ties theory [4], likely acts as a significant conduit for novel information.

One of the major findings of interest was that value ratings were low across the majority of partners. Value ratings may have been low because the network is in the early stages of development and partners may still be learning about the advantages related to collaboration and potential impacts. Ratings would be expected to increase as the network gains momentum and demonstrates increased productivity and indicators of success. As demonstrated by Long et al. [19], network connections and level of collaboration was increased significantly over the course of several years. Partner perceptions of value could be expected to increase as network impacts grow. These findings may also be applicable to other nascent KT networks. Value ratings in the early stages of formation of the collaborative may be expected to be low as partners are not yet familiar with network objectives and progress toward these goals.

Level of value ratings might also be explained by the notion that collaboration among partners in KT networks has been found to be influenced by several factors, including geographic proximity, professional similarity and past relationship history [18]. The Frayme network is not localised but engages partners from across Canada and the world. Frayme is an intermediary for many of the relationships and partners do not necessarily have functional relationships with each other. In addition, many of the network members are working within different sectors and disciplines and may not be familiar with each other’s work. This suggests that Frayme may have the opportunity to enhance value perceptions among network members by profiling individual organisations and the potential leveraging opportunities that might exist through their work. This may be a strategy that would be useful for other KT networks seeking to leverage partnerships toward more functional relationships. For example, raising funders’ awareness of organisations and programmes that fall within their philanthropic interest area might be a way to enhance connections and strengthen economic investments [22]. In addition, Long et al. [18] note that social gatherings to support relationship development would also be beneficial for building network connections. Of note, the PARTNER tool was developed to measure public health collaborations, wherein the majority of partners are working together, locally, on a more frequent basis and it might be unrealistic to expect comparable value scores within a dispersed KT network.

Another key result was that the overall density score for the Frayme network was 20.6%. This score identifies that only one-fifth of the possible connections among partners existed and reflects a relatively low level of interconnections. This suggests that there is a significant potential for the development of increased connections among existing members. Recognising that the network is largely dispersed and that not all connections would necessarily represent mutually beneficial advantages, substantial increases in density may not be feasible or represent enhanced functioning of the network.

Frayme is a KT network with a mandate to increase the uptake of IYS; as a result, the overall structure reflects a network of IYS networks. In future, it would be interesting to examine the interconnections among these localised subgroups and calculate scores based on geographic location and membership within individual IYS collaboratives. For example, most network members were engaged with each other within cooperative activities, whereby only a small subgroup described their interactions as integrated. Integrated activities would likely be more representative of a working relationship among organisations within an IYS collaborative or local public health initiative as opposed to network members participating in knowledge exchange activities and events. It may be useful for other KT networks focused on enhancing local collaborations to examine subgroup analyses to identify changes in connections among local partners. In addition, it would be interesting to examine subgroups working within similar contexts, such as rural, northern or Indigenous communities, who might stand to receive significant benefit from exchanging lessons learned with other similar contexts. KT networks with specialised partner subgroups may benefit from examining these groups individually and to identify how relationships differ in order to apply the findings to enhance outcomes with these audiences. It would be beneficial for IYS collaboratives and other networks working within youth mental health to apply SNA within evaluation in order to better understand the composition of their partners and how they are interacting in order to improve partnerships.

It is also interesting to note that value ratings were high for not only the Leadership Team members, but also the AYM and the Family Advisory. This is a promising finding recognising that the Frayme mission places youth and family perspectives at the centre of strategic development and promotes youth and family engagement within IYS as well as within system transformation efforts. Other researchers have highlighted the importance of consumer engagement [15] and, within research in other KT networks, they have identified significant impacts from consumer engagement related to supporting the development of new insights, increased motivation and enhanced research strategies [19]. Other KT networks applying a patient-oriented approach or working in youth mental health may wish to apply SNA to examine consumer group member ratings to assess the influence over partnership awareness and investment in patient-led and youth-oriented philosophy.

Research has identified that previous KT networks have been successful by building on a foundation of existing relationships and that other networks with a highly centralised structure can promote clear messaging and high-quality evidence production [19, 26]. Centralisation is often how new public health networks are established and these collaboratives move to a more distributed leadership presentation over time [22]. Frayme’s overall structure is highly centralised and many of the network members had previous relationships with members of the Leadership Team. This helped to accelerate initial mobilisation of knowledge and likely established a base of trust among members. Grimshaw [15] has described the notion of opinion leadership, whereby individuals situated at vantage points of strategic interconnection within networks have the capacity to influence peers’ behaviour on a consistent basis. According to Grimshaw, having opinion leadership “*is not a function of the individual’s formal position or status in the system; it is earned and maintained by the individual’s technical competence, social accessibility, and conformity to the systems norms*” ([15], p. 7). Recognising that the Frayme network is centralised around the Frayme secretariat and other key stakeholders, the Frayme Leadership Team, the AYM and the Family Advisory may play the role of opinion leadership and, through this role, may have an enhanced opportunity to create systems transformation through social influence. Other KT networks may benefit from identifying the most central partners within their collaboration and explore whether they have particular characteristics that might be creating an influence over the development of the network. If members demonstrate thought leadership characteristics, they can be recruited to support partnership development efforts and expand network connections. In addition, it would be beneficial to ensure that thought leaders also represent relevant diversity perspectives that are needed for a strong network such as youth and family advocates within youth mental health collaboratives.

With respect to resources, the most commonly cited contributions were information, content expertise, method expertise and network connections. Partners with these resources were strategically invited to the network and their previous work has been leveraged and mobilised to achieve Frayme’s goals. This approach was based both on the specific leadership strategy as well as the contextual environment. D’Andreta et al. [26] have described this as enactment within KT networks, whereby implementation mechanisms, context and creative governance are all involved in influencing how a network functions and creates impacts. Following a strategic approach aligns with recommendations for quality rather than quantity connections. Researchers have recommended growing networks using a strategic approach that leads to quality rather than quantity of partnership connections [19, 22, 23]. In addition, unstructured network growth can become difficult to manage and lacking in focus [19]. This approach may be applicable to other KT networks with limited resources who are looking to expand networks through strategic and in-depth relationship development to enhance specific outcomes.

Collaborative relationships may be particularly valuable in low-resource settings as partners can offset deficits by sharing resources and complementary supports. In addition, collaborations in low-resource settings can also benefit from technology in order to bridge distances among partners, support communication and to automate administrative processes. In particular, free and low cost technology, such as Canva (https://www.canva.com/), Unsplash (https://unsplash.com/), Youtube (https://www.youtube.com/) as well as a range of Google software such as Forms, Docs and Hangouts (https://about.google/intl/en/products/?tab=ch), can be used to support KT and capacity-building activities.

Finally, the analysis found that the Frayme secretariat had a large amount of non-redundant or weak ties, reflecting connections with no other adjacent connections. As such, the Frayme secretariat is uniquely positioned to act as a broker among partners. Other research has found that brokers serve unique roles in that they are able to make personal connections to support new partnerships, span inter-disciplinary and inter-sectoral boundaries and overcome power differentials within KT networks [19, 25]. KT networks interested in bridging interdisciplinary boundaries and creating new avenues for exchange of ideas might benefit from analysing network connections to identify weak ties. Partners presenting with increased weak ties may then be targeted to enhance the flow of information and strengthen partnerships.

### Limitations and future directions

There were several difficulties encountered when collecting the survey that may have limited our findings. The survey did not include a ‘don’t know’ or ‘not applicable’ option and many of the survey respondents reported that this would have helped them to better complete the questions. In addition, some partners were included as individuals, some as group representatives and some as organisation representatives. For reporting on relationships with organisations or groups, respondents noted that it was hard to differentiate between relationships with individuals within organisations and with organisations as a whole. Partners who were well connected identified that the survey was very lengthy, as they had to complete the relational questions for each network member they were working with. In addition, responses would likely vary depending on which department or representative they are collaborating with within an organisation. In addition, as individuals change roles and organisations within the network, their past histories with organisations and current relationships would change, yet their perspective would be influenced by both experiences. Finally, many respondents noted that they had limited knowledge of the nature of involvement with Frayme by other partners and had difficulty responding to the questions as a result. This is a reflection of the distributed nature of the network and highlights possible strategies to profile network members work and possible collaborative opportunities. Within other SNAs examining KT networks, researchers have noted that respondents had difficulty interpreting survey questions resulting in asymmetrical reporting of partnerships, whereby one partner reported a working relationship, which was not reported in turn by the other partner [18].

In future, it would be interesting to examine whether the network is able to support interdisciplinary connections and to identify whether novel ideas and research strategies have been created that can better examine collaborative service approaches. In addition, only key partners were invited to participate in this SNA. Going forward, it would be interesting to collect information from local IYS partners to examine relationships within IYS collaboratives and to identify how these partnerships are being formed.

## Conclusion

This study examined how relationships were formed within an international KT network designed to promote youth mental health through the integration of services. Frayme network members reported a generally high level of trust and high value ratings for key stakeholders. These findings increase understanding with respect to the mechanisms of network development and will be helpful to inform partnership development in the future. In addition, they contribute to the literature with respect to KT practice as well as the scaling of collaborative interventions within youth mental health.

## Supplementary information

**Additional file 1.** Data collected using the PARTNER tool survey and software (PARTNER_data_Apr14_2019_Anonymous).

## Data Availability

All data generated or analysed during this study are included in this published article [and its supplementary information files].

## Abbreviations

AYM: Advisory on Youth Matters (the Frayme youth advisory)
IYS: integrated youth service
KT: knowledge translation
SNA: social network analysis
